# Targeting the glymphatic system: Aβ accumulation and phototherapy strategies across different stages of Alzheimer’s disease

**DOI:** 10.1186/s40035-025-00510-8

**Published:** 2025-09-24

**Authors:** Danrui Zhao, Junting Wang, Yirui Zhu, Hao Zhang, Chenkang Ni, Zhuowen Zhao, Jingyu Dai, Rongqiao He, Guangzhi Liu, Cheng Gan, Shouzi Zhang, Zhiqian Tong

**Affiliations:** 1 Beijing Geriatric Hospital, Beijing, 100095 People’s Republic of China; 2 Westlake Univ, Sch Life Sci, Hangzhou, Zhejiang People’s Republic of China; 3 Zhejiang Provincial Clinical Research Center for Mental Disorders, The Affiliated Wenzhou Kangning Hospital, School of Mental Health, Wenzhou Medical University, Wenzhou, Zhejiang People’s Republic of China; 4 The First School of Medicine, School of Information and Engineering, Wenzhou Medical University, Wenzhou, 325000 Zhejiang People’s Republic of China; 5 Key Laboratory of Mental Health, Institute of Psychology, Chinese Academy of Sciences, Beijing, People’s Republic of China; 6 Plastic Surgery Hospital, Chinese Academy of Medical Sciences, Beijing, People’s Republic of China

**Keywords:** Alzheimer’s disease, Glymphatic system, Amyloid-β clearance, Photobiomodulation therapy, Meningeal lymphatic vessels

## Abstract

The glymphatic system serves as the brain’s clearance system. It deteriorates with age and is a significant contributor to the onset and progression of Alzheimer’s disease (AD). Modulating cerebrospinal fluid (CSF)-based clearance and targeting key components of the glymphatic system, such as aquaporin-4, can enhance amyloid-beta (Aβ) clearance. Light therapy is emerging as a potential AD treatment approach, which involves the use of visible and near-infrared light at specific wavelengths (630/680/808/850/1070 nm), photosensitive proteins, and sensory stimulation at particular frequencies (e.g., 40 Hz). This phototherapy strategy can broadly influence the intracerebral fluid dynamics, including cerebral blood flow, CSF, and interstitial fluid (ISF), as well as structures related to the glymphatic system, such as vascular endothelial cells, glial cells, and neurons. Additionally, it may directly or indirectly inhibit Aβ accumulation by modulating endogenous small molecules, thereby improving cognitive function. Our previous research demonstrated that 630-nm red light can inhibit Aβ cross-linking by clearing endogenous formaldehyde and promoting ISF drainage. Notably, Aβ accumulation exhibits distinct characteristics at different phases of AD, accompanied by varying features of glymphatic system impairment. In the early stages, deep brain regions are significantly affected, whereas in the late stages, accumulation primarily occurs in the paracentral, precentral, and postcentral cortices. Owing to the limited penetration depth of light, this may pose a challenge to the clinical efficacy of phototherapy. Therefore, different stages of AD may require tailored phototherapeutic strategies. Meanwhile, it is important to acknowledge the ongoing controversies associated with lymphovenous anastomosis, a procedure that targets the glymphatic system. Therefore, this article reviews the characteristics of glymphatic system impairment across various AD stages and the mechanisms by which effective phototherapies modulate the glymphatic system. Potential phototherapeutic strategies corresponding to different stages of Aβ accumulation are also proposed.

## Introduction

Alzheimer's disease (AD) is an age-related neurodegenerative disorder and a leading cause of dementia in the elderly. As of 2018, there were more than 50 million AD patients globally; among them, AD patients in China accounted for 25% [1]. As a characteristic pathological manifestation of AD, amyloid-beta (Aβ) deposition has been an important target for drug development over the past two decades [2, 3]. However, the development of drugs targeting Aβ secretases has faced challenges due to the blood–brain barrier (BBB) and the side effects of drugs, particularly secretase inhibitors [3, 4]. What is even more frustrating is that despite an investment of over $600 billion, more than 95% of the drugs cannot significantly improve cognitive function in classic scale tests [5]. Since the proposition of the glymphatic system as a waste clearance pathway in the brain in 2012, it has gradually become an important target for the treatment of AD [6]. Imaging evidence from AD patients has confirmed that the perivascular space (PVS) of the glymphatic system is abnormally dilated and the rate of Aβ clearance detected by arterial spin labeling (ASL) progressively decreases. More surprisingly, Aβ deposits sequentially with “prion-like” characteristics at different stages of AD [7], which is similar to the drainage direction of CSF in the brain. The characteristics of glymphatic system impairment at different stages of AD and the involvement of the glymphatic system in the progression of AD have become the next focus of AD research.

In recent years, non-invasive phototherapy has been used in the treatment of AD. In particular, the 40 Hz audiovisual stimulation therapy pioneered by Tsai et al. has entered phase III clinical trials [8]. This therapy reduces Aβ deposition by regulating the glymphatic system. Other photobiomodulation (PBM) therapies have also significantly improved the cognitive function of AD mouse models [9]. These phototherapy strategies can bypass the BBB, directly activate mitochondrial cytochrome *c* in the brain, inhibit Aβ deposition, and exert neuromodulatory effects in the deep brain through interaction with photosensitive proteins [10–13]. Therefore, they show potential application value in the treatment of AD.

## Current imaging evidence of glymphatic impairment

Since the discovery of the glymphatic system in rodents in 2012, Aβ clearance has been considered to be regulated by the glymphatic system [14]. Subsequently, Iliff, Yang and other groups, by using techniques such as dynamic contrast-enhanced magnetic resonance imaging (MRI) and time-sequenced ex vivo fluorescence imaging, obtained crucial evidence for the inhibition of the CSF–ISF exchange in rodent models of AD [15, 16]. This not only provides a methodological basis for non-invasive imaging of the human glymphatic system, but more importantly, suggests that the declined function of the human glymphatic system may be an important biomarker for predicting the occurrence and progression of AD.

In 2017, analysis of index for diffusivity along the PVS using the diffusion tensor image analysis along the perivascular space (DTI-ALPS) technique, was first proposed as an indirect and non-invasive method to evaluate the function of the human glymphatic system, and is associated with AD cognitive function and clinical manifestations [17]. Starting from DTI-ALPS, more non-invasive monitoring techniques for the glymphatic system are under development. Recently, Iliff and colleagues reported a high-resolution detection technique for the function of the glymphatic system by monitoring CSF, neural, and vascular activities, further providing evidence for whether the glymphatic system dysfunction contributes to the development of AD [18]. In this section, we will introduce classical and the latest imaging techniques for the glymphatic system and discuss imaging evidence of glymphatic system damage in AD.

### Imaging evidence of paravascular pathway impairment

Microscopic imaging combined with various fluorescent tracers and antibodies has significantly advanced our understanding of the structure of AQP4 and the flow direction of CSF in the paravascular pathway. Among them, two-photon microscopy enables real-time tracking of the dynamics of CSF in the PVS of living animals, revealing the rhythmic, pulsation-driven inflow of CSF [6, 19, 20]. Confocal microscopy has enabled high-resolution imaging of astrocytic end-feet and AQP4 [21]. When combined with tissue clearing techniques, confocal microscopy permits three-dimensional reconstruction of the whole-brain glymphatic system, which is crucial for exploring CSF drainage pathways and discovering new structures [22]. Unfortunately, these invasive imaging methods have limited use in AD patients. Therefore, we mainly discuss imaging evidence for the impaired glymphatic system in AD patients obtained through non-invasive methods such as MRI.

#### DTI-ALPS

**Fig. 1:**
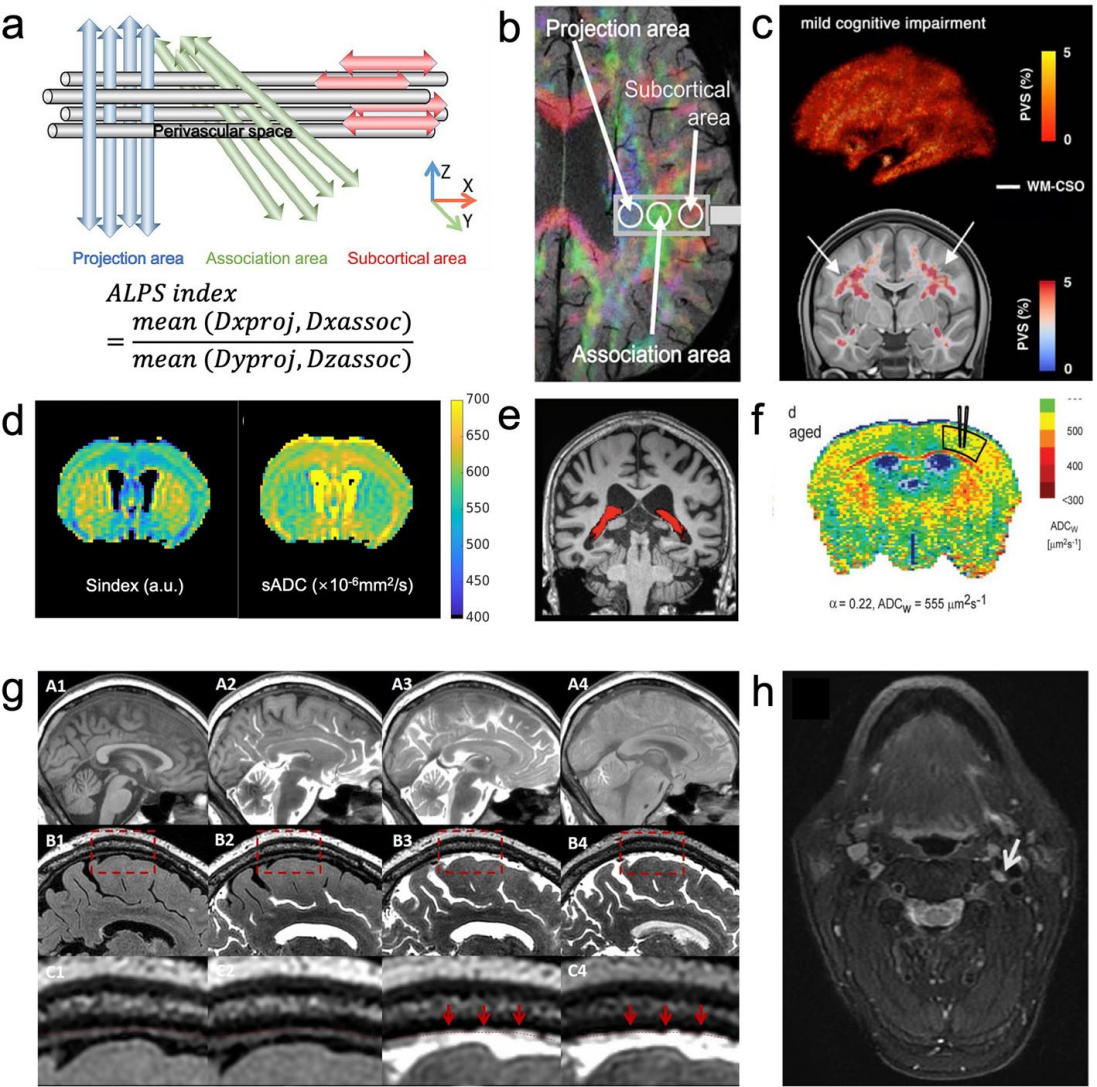
Imaging of glymphatic dysfunction in AD. **a** Schematic of DTI-ALPS. **b** Superimposed color display of DTI in an AD patient on susceptibility-weighted imaging, indicating the distribution of projection fibers (z-axis: blue), association fibers (y-axis: green), and subcortical fibers (x-axis: red). The diffusivity of the x-, y- and z-axes can be measured to calculate the ALPS index.** a** and **b** panels were reproduced from Ref. [17, 40–42] with permisson, copyright © Japanese Journal of Radiology, 2017. **c** PVS maps of an MCI patient obtained via T1-weighted and FLAIR sequences and image processing algorithms. The white arrows point to the WM-CSO. Reproduced from Ref. [28] with permission, copyright © Neurobiology of Aging, 2021. **d** Sindex maps and sADC maps of mice with inhibited APQ4 channels obtained via DW-EPI. Reproduced from Ref. [30] with permission, copyright © PLoS One, 2020. **e** 3.0-T brain MRI of the choroid plexus (CP) volume (red) in an AD patient. Reproduced from Ref. [32] with permission, copyright © Radiology, 2022. **f** An ADC_W_ map of the brain of an APP23 female mouse obtained via the DW-MRI technique. The mean value of the ADC_W_ was calculated in the areas indicated. Reproduced from Ref. [35] with permission, copyright © Proc. Natl. Acad. Sci. U.S.A., 2005. **g** Images of putative meningeal lymphatic vessels (pMLVs) in elderly patients acquired via 3D-T1 imaging and 3D-T2-FLAIR imaging. Red wireframes and red arrows are pMLVs. **h** Images of dcLNs in an older adult via neck T1-fat-suppression imaging. **g** and **h** panels were reproduced from Ref. [36] with permission, copyright © Ann Neurol, 2020

In 2017, Taoka et al. developed the DTI-ALPS technique for the first noninvasive assessment of human brain glymphatic system function [17]. DTI-ALPS is based on diffusion tensor imaging (DTI), an MRI technique that measures diffusivity in the direction of projection fibers, contact fibers, and perivascular interstitial spaces of the medullary veins in the plane of the lateral ventricle body; thus, the ALPS index can be calculated (Fig. 1a, b). The ALPS index positively correlates with the functional strength of the glymphatic system [17, 23, 24]. It decreases progressively through different stages of AD and can be used to predict the risk of clinical progression in AD patients [25]. In particular, the basal ganglia (BG)-PVS volume fraction negatively correlates with the DTI-ALPS index [23].

#### PVS segmentation volume

High-field MRI enables direct visualization of PVS morphology [26]. After acquiring the interpolated coronal T2-weighted MR images, the automated segmentation and volume calculation of PVS is performed with the deep learning model U-Net. In addition, the use of T1-weighted and fluid attenuated inversion recovery (FLAIR) sequences and the multimodal alignment of the two, combined with image processing algorithms (such as the adaptive nonlocal mean filtering technique and Frangi filters), enable accurate quantification of the PVS volume fraction [27, 28].

PVS volume inversely correlates with cognitive function in AD patients [29]. In particular, the volume of PVS in the centrum semiovale is significantly enlarged in AD patients compared to the control group, and the volume of PVS is negatively correlated with cognitive function scores. PVS volume assessment in the centrum semiovale is an early imaging biomarker of AD [29]. Patients with mild cognitive impairment (MCI) have higher PVS volume scores in the centrum semiovale of the white matter (predominantly present in women) (Fig. 1c) and lower PVS volume scores in the anterior superior medial temporal lobe (asMTL) compared to controls [28]. The changes in PVS in the asMTL may represent an early feature of AD pathology.

#### AQP4 imaging

AQP4 affects the CSF-ISF priming rate. Diffusion-weighted echo planar imaging (DW-EPI) is a diffusion MRI technique based on an 11.7T ultrahigh field scanner and a multi-b-value design to noninvasively image AQP4. After inhibition of astrocyte AQP4 channels, DW-EPI revealed a decrease in the diffusion marker S-index and an increase in the water shifted apparent diffusion coefficient (sADC) (Fig. 1d). DW-EPI for noninvasive monitoring of AQP4 channel activity [30] may be a potential indicator for assessing AD severity.

Water-exchange dynamic-contrast-enhanced MRI (DCE-MRI) can measure the transmembrane water-efflux rate, a sensitive biomarker of AQP4, and thus dynamically monitor AQP4 expression. Water-exchange DCE-MRI modifies the scanning parameters of conventional DCE-MRI with higher sensitivity and specificity for transmembrane water exchange [31].

#### Volume imaging of the choroid plexus (CP)

In a retrospective study by Choi et al., DCE-MRI and quantitative susceptibility mapping revealed that the CP volume was significantly increased in patients with AD and correlated with the severity of cognitive impairment [32]. The possible mechanism is that the increased CP volume may be related to interstitial fibrosis, vascular thickening or inflammation, ultimately leading to impaired CSF clearance function. The technique uses 3.0-T MRI with deep learning–based segmentation, enabling accurate measurement of the CP volume (Fig. 1e).

### Extracellular space (ECS) impairment in AD

ECS imaging is mostly performed using optical methods such as super-resolution shadow imaging (SUSHI) [33] and super-resolution imaging [34]. However, there are limitations in the resolution and the depth of penetration of optical imaging techniques. MRI techniques with high tissue penetration are preferable when deep structures are involved.

The diffusion-weighted MR (DW-MR) technique detects the Brownian motion of water molecules in tissues by applying a diffusion-sensitive gradient magnetic field, selecting the stimulated echo sequence and combining it with a multi-b-value diffusion gradient. The degree to which the diffusion of water molecules is limited can influence the magnetic resonance signal intensity, allowing for the calculation of the apparent diffusion coefficient of water (ADC_W_). A previous study reported that the ADC_W_ of aged female APP23 transgenic mice was significantly greater than that of controls of the same age, suggesting that their ECS expanded (Fig. 1f) [35].

### Brain lymphatic vessel

CSF drainage and clearance of meningeal lymphatic vessels (mLVs) can be assessed by intrathecal injection of gadolinium contrast agent followed by dynamic tracking of the contrast agent via three-dimensional T1-weighted imaging. In a previous study, this method was combined with high-resolution T2 FLAIR to suppress CSF signals and visualize meningeal lymphatics (Fig. 1g). The results revealed that the clearance function of both the glymphatic system and the meningeal lymphatics was negatively correlated with age, with a significant delay in clearance in elderly patients [36].

In a more recent study, DCE-MRI was used to assess the clearance function of putative mLVs by observing signal changes in the parasagittal dura; 2D FLAIR sequences were used to quantitatively assess the intensity of inferior frontal sulcus hyperintensity (IFSH) by suppressing CSF signals and highlighting high-signal lesions in the brain parenchyma. The signal intensity of IFSH was found to be significantly correlated with impaired clearance of mLVs, and the visibility of IFSH on 2D FLAIR may serve as an indicator for assessing clearance dysfunction in mLVs [37]. IFSH signal intensity assessed by 2D FLAIR may serve as an early imaging marker for AD.

### Brain–peripheral lymphatic connectivity

Deep cervical lymph nodes (dcLNs) are the main bridge for CSF drainage to the periphery. Stereoscopic photoacoustic microscopy with deep imaging capabilities showed that in the AD mouse model, the drainage volume of mLVs to the dcLNs decreases by 70% and further declines as AD progresses [38]. Below, we will review possible methods for central-deep cervical lymph node imaging.

#### Neck T1-fat-suppression imaging

T1-fat-suppression imaging suppresses the fat signal and highlights the enhancement signal of the dcLNs on the basis of T1-weighted imaging. After intrathecal injection of gadolinium dimeglumine contrast agent, the drainage of the contrast agent from putative mLVs to the dcLNs can be assessed via T1 fat-suppressed imaging of the neck (Fig. 1h) [36].

#### Spoiled gradient echo (SPGR) MRI imaging

The variable-flip angle 3D spoiled gradient echo (VFA-SPGR) T1 mapping technique enables single snapshot imaging without the need for a baseline scan. The drainage efficiency of transcervical lymph nodes has been quantified in mice via SPGR sequence MRI after injection of gadolinium contrast agent (Gd-DOTA) into the CSF. Under anesthesia, the T1 values of the dcLNs are significantly lower and decreased more than submandibular lymph nodes (smLNs), suggesting that the dcLN is a major drainage target of the glymphatic system [39].

#### Dynamic contrast-enhanced MR lymphangiography (DCE-MRL)

DCE-MRL, which uses sequentially precontrast static sequences, dynamic enhancement sequences, and high-resolution T1 sequences combined with respiratory gating and fat suppression techniques, has high temporal and spatial resolution and dynamic imaging capability. DCE-MRL has been clinically applied in the diagnosis of congenital lymphatic malformations, lymphatic dynamics abnormalities, lymphatic leakage, and other diseases.

Future applications of DCE-MRL technology for deep cervical lymphatic vessel imaging are expected.

## Core and potential biomarkers at different stages of AD

Currently, biological disease definition has become the consensus framework for neurodegenerative disorders, supplementing clinical symptom assessment [43]. In AD, core proteins such as Aβ and tau are directly associated with neuropathology. In 2024, the National Institute on Aging and the Alzheimer’s Association (USA) revised the diagnostic and staging criteria for AD. The biological staging based on the A (Aβ proteinopathy), T (AD tau proteinopathy), and N (neurodegeneration) classification was further clarified. Specifically, according to the positron emission tomography (PET) and fluid biomarker results, AD is divided into four biological stages: initial, early, intermediate, and advanced. It is worth noting that the biological staging, as a supplement to clinical diagnosis, cannot completely replace the classic clinical staging. Therefore, in this article, we retain description of the classic clinical staging and attempt to integrate both frameworks with glymphatic impairment characteristics.

AD is a spectrum of continuous diseases (Alzheimer continuum) whose clinical symptoms are influenced by interindividual differences in neuropathology and cognitive reserve and do not strictly correspond to the degree of accumulation of the biomarkers mentioned above. However, it is still possible to stage typical clinical progression in correspondence with biomarkers. AD patients experience a long asymptomatic period (preclinical, stage 1) showing only genetic and biomarker abnormalities, with CSF levels of Aβ and tau beginning to rise, accompanied by sleep disorders [44]. As the disease progresses, patients first develop memory impairment and gradually progress to MCI (stage 3) [45], with involvement of the hippocampus, insula, amygdala, and other brain regions [46–51]. Dementia (stage 4–6), in which the patients suffer severe memory impairment and loses all abilities to care for themselves [52]. Patients in this period have large amounts of Aβ deposits and neuronal tangles in brain regions including the cerebellum [53].

In our previous studies, nonspecific factors such as formaldehyde (FA) and cytokines are also associated with the development of AD [54]. In addition, recent studies have shown that the CSF YWHAG:NPTX synaptic protein ratio may be a new reliable biomarker to predict the onset and progression of AD [55].

### Core toxic protein accumulation

#### Aβ

**Fig. 2:**
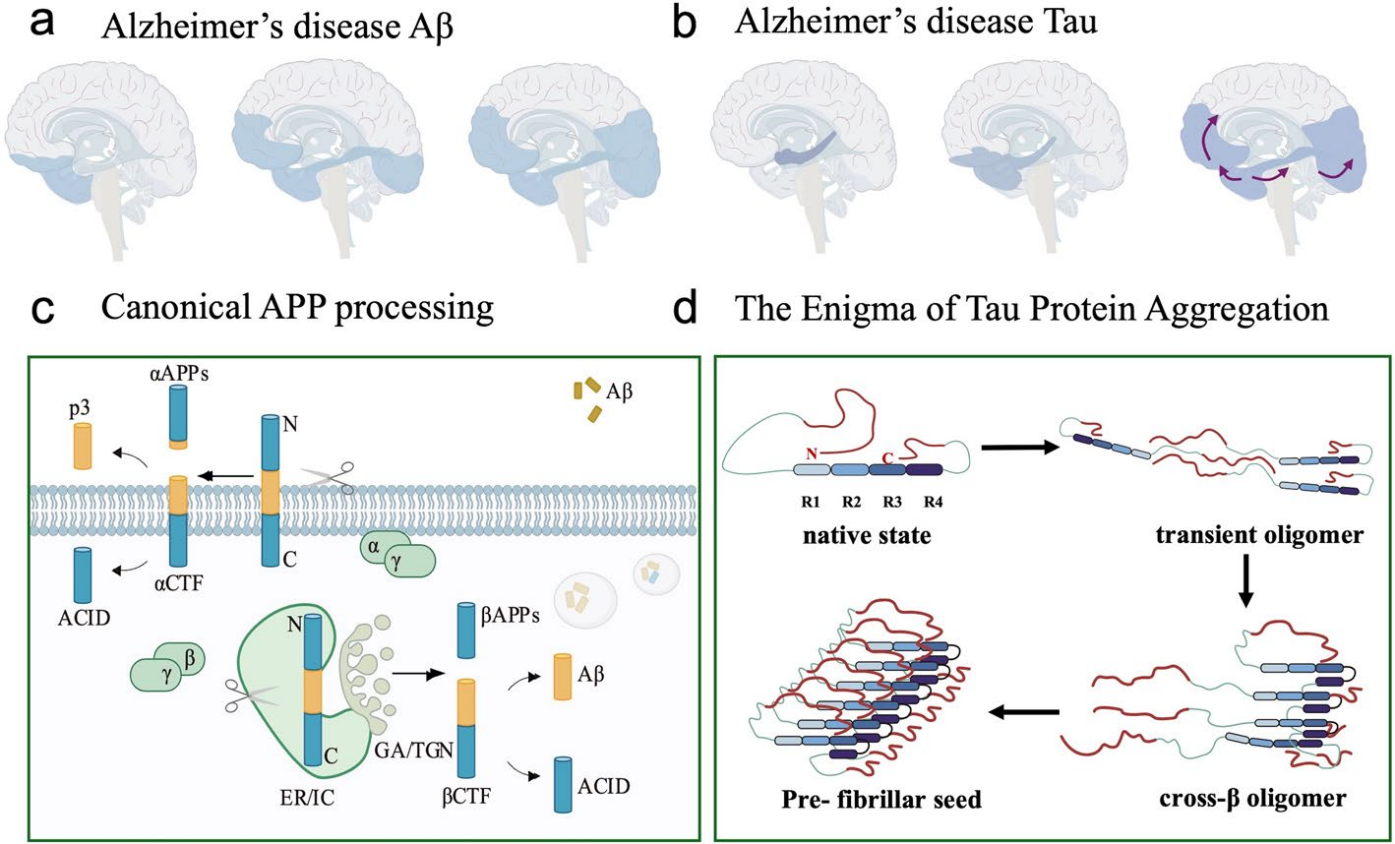
Sequential accumulation of Aβ and tau. **a, b** Beginning and development of Aβ and tau accumulation. Reproduced from Ref. [65] with permission, copyright © The American Association for the Advancement of Science., 2020. **c** Aβ generation from APP. **d** A possible mechanism of tau misfolding from the paperclip conformation to prefibrillar seeds

Aβ deposition is an important pathologic change in the development of AD. The CSF Aβ42 level is significantly changed 18 years before diagnosis and the Aβ42/40 ratio changed significantly 14 years before diagnosis. Aβ deposition begins in deeper parts of the brain, and then progresses to the whole brain (Fig. 2a). Mattsson et al. investigated regions of the brain where Aβ is deposited in AD patients via PET. The results revealed that Aβ amyloid deposition in early AD patients mainly involves five deep brain regions: the precuneus, posterior cingulate gyrus, cingulate isthmus, insula, and orbital frontal cortex. The lingual gyrus, perisylvian sulcus, paracentral lobule, anterior central gyrus, and posterior central gyrus are main brain regions involved in late Aβ deposition. The remaining brain regions are categorized as the main regions involved in the middle stage of AD [56]. Thal et al. categorized AD into five phases based on Aβ deposition via autopsies of AD patients. In phase 1, Aβ accumulation is detected in frontal, parietal, temporal, or occipital neocortex. As the disease progresses, Aβ accumulation gradually involves the cortex, mesencephalic nuclei, striatum, and other structures until the whole brain becomes involved [53].

Changes in Aβ levels in plasma and CSF are important in the progression of AD. Aβ42 levels in the CSF progressively decline during AD progression, whereas Aβ40 levels remain essentially unchanged, making the Aβ42/Aβ40 ratio a reliable predictor of AD [57, 58].

*Mechanisms of Aβ accumulation* The clearance of Aβ relies on both enzymatic and glymphatic drainage pathways, and the development of Aβ accumulation in AD patients is closely related to impaired clearance. Under normal conditions, Aβ amyloids can be degraded by enzymes or excreted from the brain via receptor-mediated excretion [59, 60]. However, in AD, the level and activity of Aβ catabolism-related enzymes are significantly reduced [61, 62], resulting in a failure of timely Aβ degradation. In addition, the level of LDL receptor–related protein-1 mediating Aβ transport is also reduced [63, 64]. This dual impairment drives Aβ accumulation.

Aβ protein self-replication and propagation are also possible mechanisms leading to Aβ deposition. Recent studies have shown that Aβ has a prion-like mode of transmission. Fritschi et al*.* demonstrated that extracts from formalin-fixed (two years) Aβ-containing brain tissue could still induce Aβ deposition when injected into the dorsal hippocampus of mice [66].

*Pathologic effects of Aβ* Aβ has toxic effects on neurons, glial cells, cerebral blood vessels, and peripheral cells, and is associated with neurological damage, tau protein hyperphosphorylation and glymphatic impairment in AD patients. Aβ oligomers have a direct toxic effect on neurons [67], leading to an increase in toxic substrate levels to induce oxidative stress [68] and thus damage mitochondria [69]. Aβ can also induce tau protein hyperphosphorylation and the formation of neurofibrillary tangles (NFTs) [70]. Nortley et al. demonstrated that Aβ amyloid can stimulate microglia to produce reactive oxygen species (ROS), leading to pericyte contraction, which is closely related to the cerebral blood flow (CBF) decline in AD patients [71]. Activated microglia also release inflammatory factors triggering neuroinflammation [72]. Insoluble fiber Aβ deposition in the walls of cerebral capillaries and arteries leads to cerebral amyloid angiopathy, where the elasticity of the vessel wall decreases and hemorrhage is likely to occur [73]. This also impairs the drainage function of the glymphatic system [74].

#### Tau

According to the Alzheimer's Association Workgroup latest guidelines [43], the core abnormal tau protein isoforms in CSF and plasma are divided into two categories. The tau isoforms belonging to Core 1 biomarkers appear earlier than clinical symptoms and can be used for preclinical diagnosis. The tau isoforms belonging to Core 2 biomarkers appear later in AD patients but are more closely associated with symptoms in AD patients [43].

NFTs are an important pathological change in the brains of AD patients [75], composed mainly of the tau protein [76]. Tau protein aggregation starts from the deep part of the brain and gradually involves the whole brain, resulting in a deep-to-shallow and local-to-whole lesion process (Fig. 2b). Braak et al. categorized AD into 6 stages on the basis of tau protein aggregation according to the autopsy results of AD patients, which starts from the transcortical internal olfactory cortex (stage I), sequentially involving the internal olfactory cortex and hippocampus (stage II), the neocortex (stage III), the associative cortex (stages IV and V), and finally the cortex (stage VI) to develop into a whole-brain lesion [77, 78].

Braak staging is the dominant doctrine of AD staging, and the use of PET technology has led to new variations in Braak staging as well. A series of PET studies have shown that the involvement of the internal olfactory cortex can also occur in stage I. Involvement of the hippocampus remains a hallmark of stage II, whereas stage III is further refined to include the amygdala, parahippocampal gyrus, fusiform gyrus, and lingual gyrus, with the caudal and rostral anterior cingulate gyrus thought to be the area of involvement in stage V. Stage VI is identified as the involvement of paracentral, postcentral, and precentral gyri [79, 80]. Overall, Braak staging remains highly informative, and imaging findings are generally consistent with this staging.

Plasma and CSF levels of p-tau also reflect the course of AD. The Core 1 proteins of the guidelines, p-tau217, p-tau181, and p-tau231, appear abnormal in plasma and CSF well before the onset of tau aggregation [43]. CSF and plasma levels of p-tau181 are significantly elevated in AD patients, and the p-tau181/np-tau181 (non-phosphorylated-tau181) ratio responds well to Aβ deposition [81]. Recent studies have shown that p-tau217 is superior to p-tau181 in the prediction and diagnosis of AD [82], which provides new ideas for the preclinical diagnosis of AD. MTBR-tau243 is categorized as a Core 2 protein in the guideline [43]. Recent studies have shown that this isoform responds well to tau aggregation within the brain, correlating significantly with tau PET signals [83].

*Mechanism of tau aggregation and Aβ interaction* Tau proteins are microtubule proteins. Their aggregation involves hyperphosphorylation and impaired clearance. Elevated Aβ concentrations exacerbate the hyperphosphorylation of tau proteins and formation of tau oligomers, which promotes their propagation within the brain, leading to NFT lesions. Tau protein aggregation results from misfolding, and hyperphosphorylation is a prerequisite for misfolding to occur [84]. Soluble tau oligomers can propagate in a “prion-like” manner across neuronal synapses, inducing additional misfolding (Fig. 2d) [85]. In vitro studies in cell culture demonstrated that tau oligomers propagate across neurons via exosomes [86]. Tau removal is dependent mainly on the glymphatic system of the brain [14]. Animal experiments have demonstrated that AQP4 in the astrocyte endfeet mainly mediates tau removal [87]. Clinical studies have confirmed impairment of the glymphatic system in AD patients [88], which may be the main reason for the impaired clearance of tau protein.

Aβ plays an important role in the initiation and development of tau accumulation. Aβ accelerates tau hyperphosphorylation and induces tau oligomerization [89]. It has been suggested that Aβ leads to initial tau accumulation via the entorhinal cortex by inducing overexcitation of the default mode network [90]. Microglial activation and astrocyte proliferation secondary to elevated Aβ have also been implicated in the induction of tau accumulation [91, 92]. Aβ can activate CDK-5 and GSK-3β to exacerbate the hyperphosphorylation of tau [93, 94]. Aβ also promotes the cleavage of tau proteins by cysteine hydrolases to produce short-truncated tau, which is more susceptible to form oligomers [95]. Tau proteins can also synergize with Aβ to cause damage to glial cells, triggering neuroinflammation and disrupting the BBB [89]. In vitro cellular experiments demonstrated that tau protein can trigger the release of inflammatory mediators through activation of the cGAS-STING pathway [96] and that hyperphosphorylated tau can accumulate in astrocytes and impair astrocyte activity [97]. Tau determines the toxic effects of Aβ, and Aβ toxicity is significantly reduced in the brains of tau-deficient mice [98].

*Pathologic roles of tau proteins* Tau proteins have toxic effects on neurons and glial cells, associated with neuronal damage and neuroinflammation in AD patients. Hyperphosphorylation of tau leads to a loss of its role in DNA protection, resulting in increased susceptibility of neuronal DNA to damage [99]. Tau can also cause damage to mitochondria, disrupting neuronal energy metabolism [100]. The damaging effects of tau on neurons are also associated with its negative impact on synaptic plasticity, microtubule assembly and cytoskeletal stability [101].

There is growing evidence that tau proteins can damage the glymphatic system. Tau aggregation in wakefulness-promoting regions can interfere with sleep [102], leading to sleep disorders and glymphatic dysfunction [103]. Also, tau accumulation in astrocytes reduces AQP4 expression [104], which together contribute to the impairment of the cerebral lymphatic system in AD patients.

### Imaging of toxic proteins in AD

Aβ plaques and NFTs formed by hyperphosphorylated tau proteins are core pathological features of AD. Aβ deposition first occurs in the precuneus and then spreads throughout the cerebral cortex [105]. Tau proteins preferentially deposit in the internal olfactory cortex and extend to the temporoparietal lobe with disease progression [106]. Aβ in plasma and CSF are primarily detected by ELISA kits, and those located in brain regions are primarily visualized via PET. Aβ and tau PET imaging can directly visualize the distribution of deposits in the brain and is the core diagnostic basis recommended by the NIA-AA IWG guidelines [43]. The classical and novel tracers for Aβ PET and tau PET are listed Table 1. Other visualization techniques for Aβ and tau proteins are listed in Table 2.

**Table 1:** PET tracers for Aβ and tau

	Tracer	*Pros*	*Cons*
Aβ PET	^11^C-PiB [107, 108]	• High sensitivity and specificity • Validated as a benchmark	• Short half-life • Limited clinical utility
	^18^F-Florbetapir [109, 110]	• FDA/EMA-approved • Standardized clinical procedures	• Narrow SUVR dynamic range • Higher nonspecific white matter binding
	^18^F-Flutemetamol [111, 112]	• FDA/EMA-approved • High diagnostic accuracy	• Lower signal-to-noise ratio • Scanner-dependent SUVR variability
	^18^F-Florbetaben [113, 114]	• FDA/EMA-approved • Effective in mixed pathologies • Centiloid threshold validated	• Narrower dynamic range • Strict imaging window
	^18^F-NAV4694 [115]	• Detects lower levels of cortical Aβ in the early stages of AD	• Not yet FDA-approved
Tau PET	^18^F-AV1451 [116]	• High specific binding • FDA-approved	• Detects lower levels of cortical Aβ in the early stages of AD
	^18^F-THK5351 [117]	• High contrast • Wide dynamic range	• Nonspecific white matter binding • Requires MAO-B inhibitor validation
	^11^C-PBB3 [118]	• Binds multiple tau conformers • Research utility	• Short half-life • Choroid plexus interference
	^18^F-MK6240 [119]	• Strongly binds to neurofibrillary tangles • Rapid imaging	• No binding to non-AD tau • Off-target binding to neuromelanin- and melanin-containing cells
	^18^F-PI2620 [120]	• Broad tau affinity • Stable quantification	• Limited pathological validation • Scanner-dependent standardization

**Table 2:** Other imaging methods for detecting Aβ and tau

Imaging method	*Pros*	*Cons*
MALDI-MSI [121]	• High spatial resolution and molecular specificity • No requirement for radioactive tracers • Multimodal analytical capability	• Dependence on ex vivo tissue • Static analysis constraints • Technical complexity
Curcumin fluorescence imaging [122–124]	• Direct labeling of Aβ plaques • Dynamic correlations with cognitive decline	• Low bioavailability
Near-Infrared fluorescence imaging [125]	• High spatiotemporal resolution • Real-time dynamic observation	• Human application limitation

### Potential biomarkers in AD

Inflammation is a common alteration in neurodegenerative diseases, including AD. Animal studies have demonstrated that the induction of peripheral inflammation can exacerbate the development of AD [126]. Clinical studies have confirmed worse cognitive performance in AD patients with chronic peripheral inflammation [127]. Peripheral inflammation prompts immune cells to infiltrate the central nervous system (CNS) and activate microglia to induce central inflammation and exacerbate Aβ deposition [128]. As mentioned above, Aβ and tau can also activate microglia and astrocytes, promoting the release of inflammatory factors and triggering central inflammation [129]. In a clinical study, the levels of interleukin (IL)-1β, IL-6 and complement C3 increased significantly with the progression of AD [130]. MRI confirmed that the onset of brain inflammation occurs in conjunction with the accumulation of Aβ and tau [7].

Hyperactivation and aggregation of microglia are also an additional nonspecific AD pathology. In the early stages of AD, microglia congregate at Aβ plaques [131] and phagocytose Aβ to play a protective role [132]. As the disease progresses, microglia are overactivated and release large amounts of inflammatory factors that trigger central inflammation, causing damage to the CNS [133].

Reactive astrocytes are a common pathologic alteration in AD patients, and these cells may undergo cellular hypertrophy because of the overexpression of cytoskeleton proteins such as glial fibrillary acidic protein (GFAP) [134]. Pathologic changes in reactive astrocytes are caused by inflammatory factors released by microglia. The diseased astrocytes lose their original function and can cause damage to neurons [133].

### Emerging stage-specific biomarkers in AD

By analyzing the CSF of AD patients, a U.S. research team reported that YWHAG:NPTX2 can respond to cognitive decline during AD progression [55]. The YWHAG protein, belonging to the 14–3-3 family of proteins, is closely related to basic cellular functions [135], and shows elevated levels with tau pathology [136]. NPTX2, a secreted synaptic protein critical for neurotransmission, demonstrates reduced levels that directly associate with synaptic dysfunction in AD [137].

## Glymphatic impairment at different stages of AD

**Fig. 3:**
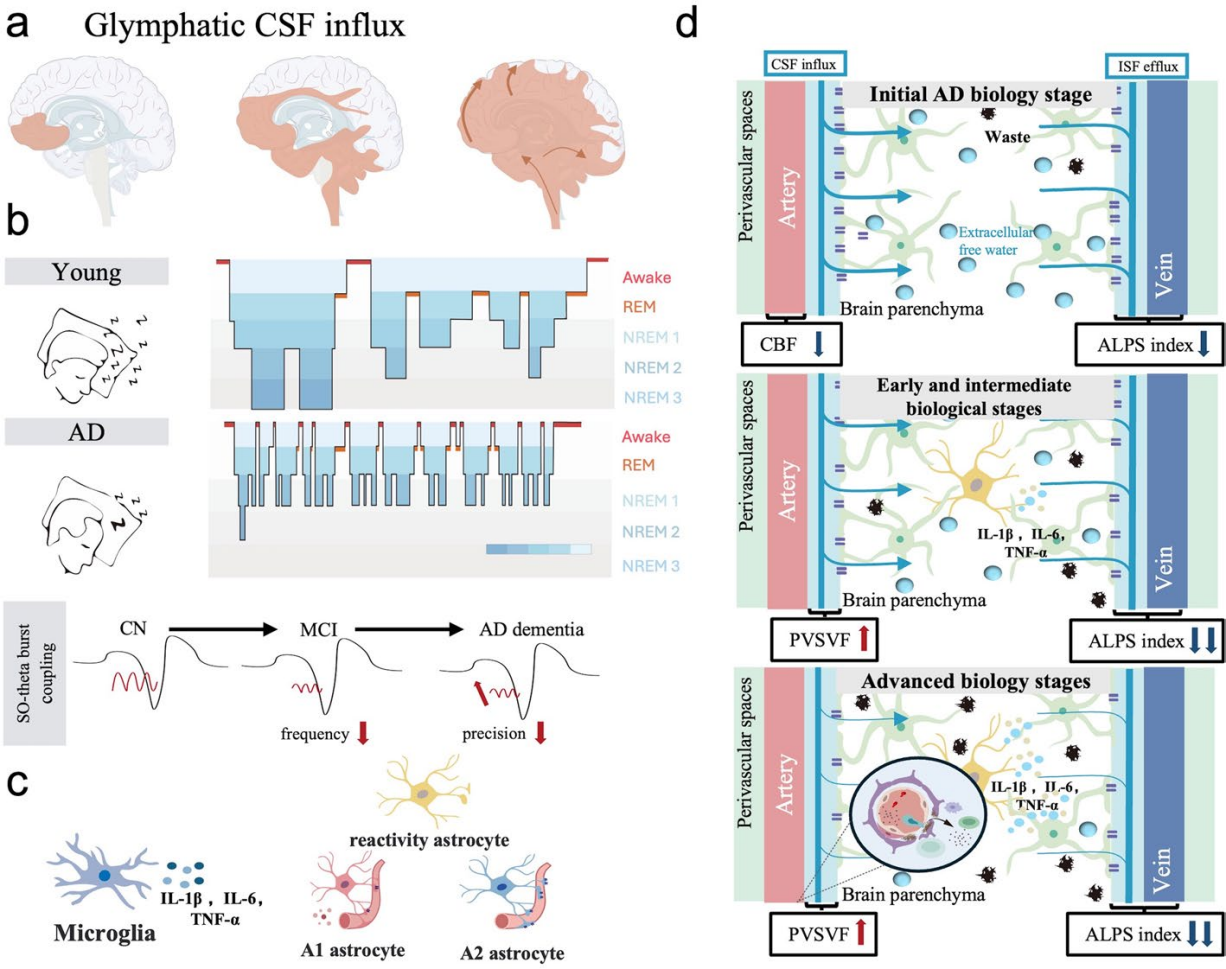
Glymphatic impairment at different stages of AD. **a** The drainage direction of CSF in the lymphatic system of the human brain is consistent with the deposition direction of Aβ and tau. Reproduced from Ref. [65] with permission, copyright © The American Association for the Advancement of Science., 2020*.* **b** Sleep fragmentation in AD patients. The NREM stage is significantly reduced and progressively disrupted during the progression of AD. **c** Changes in glial cells in patients with AD dementia. The continuous activation of microglia causes chronic inflammation, and astrocytes transform into type A1 astrocytes with less distributed AQP4. **d** The function and structure of the perivascular space are disrupted in the three pathological stages of AD

Numerous animal models have demonstrated that impairment of the glymphatic system leads to impaired core protein (Aβ, tau) clearance in AD [87]. More direct evidence is that the ALPS index decreases significantly in patients with early AD and progressively decreases with disease progression. ALPS index is also a potential biomarker for changes in Aβ42 level earlier than detection of changes in CSF Aβ42. A lower ALPS index is associated with a more rapid shift in Aβ positivity on PET and a greater risk of clinical progression, cognitive dysfunction, and brain atrophy [25]. Recent studies suggest that Aβ or tau propagates in a prion-like manner from deep brain regions toward the cortex, mirroring CSF drainage in the brain (Fig. 3a). In particular, the accumulation of low-molecular-weight CSF contrast agents (< 1 kDa) supports the idea that larger proteins are also trapped in the curved extracellular space of the deep brain [15, 65, 138]. However, there is still a lack of support from more longitudinal studies on the current characterization of dysfunction and structural damage of the glymphatic system at different stages of AD and the causal relationship with the accumulation of the core AD protein. Here, we summarize the characteristics and possible mechanisms of the glymphatic system in the various pathological stages of AD progression.

### Paravascular pathway

#### Sleep disturbance in the initial AD biology stage

The paravascular pathway clears core AD proteins, such as Aβ, primarily during sleep; however, nearly half of older adults over the age of 60 have sleep disorders [139], and more than 15% of AD cases are attributed to sleep disorders [140]. Sleep disorders are a risk factor for the development of AD; both sleep disorders and the risk of AD significantly increase with age [141]. A meta-analysis suggested that patients with sleep disorders are 3.78 times more prone to progressing to preclinical AD than healthy individuals [140]. Longitudinal changes in cognitive function in preclinical, early AD are nonlinearly related to total sleep time, non-REM (NREM) and REM sleep time, sleep efficiency, and NREM slow-wave activity [142]. In particular, reduced slow-wave sleep phases (stage 3) and disrupted circadian (sleep/wake) patterns are more prominent in AD patients (Fig. 3b) [143]. Either too low or too high a < 1 Hz slow-wave activity in NREM sleep phase is associated with significant declines in the PACC (Preclinical Alzheimer Cognitive Composite) score [142, 144].

The sleep disturbances in preclinical AD described above, especially abnormal NREM slow-wave activity, may contribute to the dysfunction of the paravascular pathway in the glymphatic system, further leading to accumulation of Aβ and tau. Among other factors, a reduction in total sleep duration significantly inhibits CSF influx and outflow through the paravascular pathway, further leading to a stagnation of ISF flow. This not only directly reduces the clearance of core AD pathogenic proteins but also creates favorable conditions for deposition in the ECS. Studies have shown that Aβ in human CSF is lowest in the early morning, but a night of acute sleep deprivation removes this clearance effect, and PET imaging reveals a significant increase in Aβ in the right hippocampus and thalamus [145, 146]. In addition, the Aβ burden in the precuneus (the brain region where Aβ is deposited in early AD stages) is negatively correlated with reported nighttime sleep duration. Moreover, the lack of slow-wave activity in NREM stage 3 leads to decreased expression and loss of polarization of AQP4 around the paravascular pathway, which likewise increases Aβ deposition [65]. A significant decrease in the DTI-ALPS index in patients with REM sleep disorders [147] may also be associated with early AD progression. In addition, CBF has been recognized as a biomarker to distinguish preclinical AD individuals from normal aging individuals. Decreased CBF also leads to decreased CSF drainage, facilitating progression to MCI.

Notably, recent studies have shown that the norepinephrine fluctuations in NREM modulate cerebral vasodilation and promote clearance via the paravascular pathway [148]. However, structural fragmentation of the NREM and dysfunction of noradrenergic nuclei such as the locus coeruleus in individuals with aging and AD are potentially involved in early sleep disorders as well as in the progression of AD [149]. Nevertheless, this hypothesis still lacks more direct clinical evidence.

#### Early and intermediate biological stages

According to the latest NIA-AA guidelines, the early and intermediate biological stages represent a transitional state before PET tau is highly accumulated in the cortex, corresponding to the typical clinical manifestations of subjective cognitive dysfunction and mild cognitive dysfunction, respectively. During this predementia transition phase, tau PET uptake from the inner olfactory cortex to cortical moderation, significant decline in reactive astrocyte-mediated ALPS, and microglia-mediated neuroinflammation, are prominent features and possible targets for intervention.

MRI studies have revealed a progressive and significant decline in the DTI-ALPS index from the preclinical stage of cognitively normal A^+^ to the MCI stage. This decline positively correlates with decline of memory and executive functioning. Aβ aggregation and brain atrophy are thought to mediate this correlation [150, 151]. Moreover, imaging studies have visualized expanded PVS space with an abnormal CP volume. This finding suggests that, unlike the slow decline in ALPS during the preclinical AD phase, impaired CSF drainage is more pronounced during AD progression, further increasing the rate of Aβ deposition. The possible mechanism is that PVS structures such as astrocytes are damaged and morphologically altered in MCI, exacerbating CSF drainage disturbances. Plasma GFAP, an astrocyte activation marker, has been used for the detection of early AD and promotes the progression of Aβ. Furthermore, elevated GFAP levels in the blood promote the progression of Aβ plaques and neurofibrillary tau tangles in later pathological stages [152]. Notably, in a streptozotocin-induced AD rat model, high-intensity intermittent training converted astrocytes to a neuroprotective A2 phenotype with AQP4 polarization, promoting Aβ and p-tau clearance (Fig. 3c) [153]. However, due to the lack of animal models of MCI, the specific molecular mechanisms involved remain unclear.

At the same time, PET imaging evidence suggests that tau tangles are first deposited in compromised paravascular pathways within the entorhinal cortex [154, 155]. However, evidence from longitudinal studies is still lacking to support a causal relationship between the paravascular pathway and the interstitial propagation of tau PET deposits in the brain.

#### Advanced biology stage

In the advanced AD biology stage, Aβ and tau accumulate throughout the brain, and patients present with dementia. Concurrently, the structure of the PVS is irreversibly damaged, resulting in vascular endothelial damage, BBB disruption, pericyte disorders, and AQP4 depolarization. Significant disruption of circadian rhythms also occurs [156]. Consequently, the ALPS index further decreases rapidly [25], leading to rapid accumulation of Aβ and tau throughout the cerebral cortex. At this stage, neuronal degeneration and cognitive dysfunction progress rapidly in cognitive-related cortex.

### Progress of the ECS deposit

Although Aβ and tau are produced intracellularly and are ultimately deposited in the ECS, there is a vicious cycle between impaired drainage of ECS ISF and Aβ deposition in AD [54]. Animal experiments revealed a significant increase in ECS volume and a decrease in the diffusion coefficient in AD mice, leading to impaired ISF clearance. Combined with decreased CSF influx and efflux in the dysfunctional paravascular pathway, this impairment results in further Aβ deposition [35].

Numerous studies have investigated the regulation of ISF Aβ/tau by the sleep‒wake cycle and the role of extracellular matrix (ECM) disruption in promoting Aβ deposition. In mice, tau in the ISF is increased by 100% during chronic sleep deprivation compared with sleep [157]. Moreover, ECM remodeling enhances the autophagy-lysosomal pathway in astrocytes, activates the astrocyte phagocytosis receptor MERTK, and facilitates astrocyte vesicle recycling, which mediates Aβ clearance and attenuates AD pathology [158]. These findings suggest that the ECS plays a central role in mediating Aβ deposition and AD progression, making it a potential therapeutic target. However, it remains unclear whether ECS dysfunction and impaired ISF drainage contribute to the spatiotemporal sequence of Aβ deposition.

**Fig. 4:**
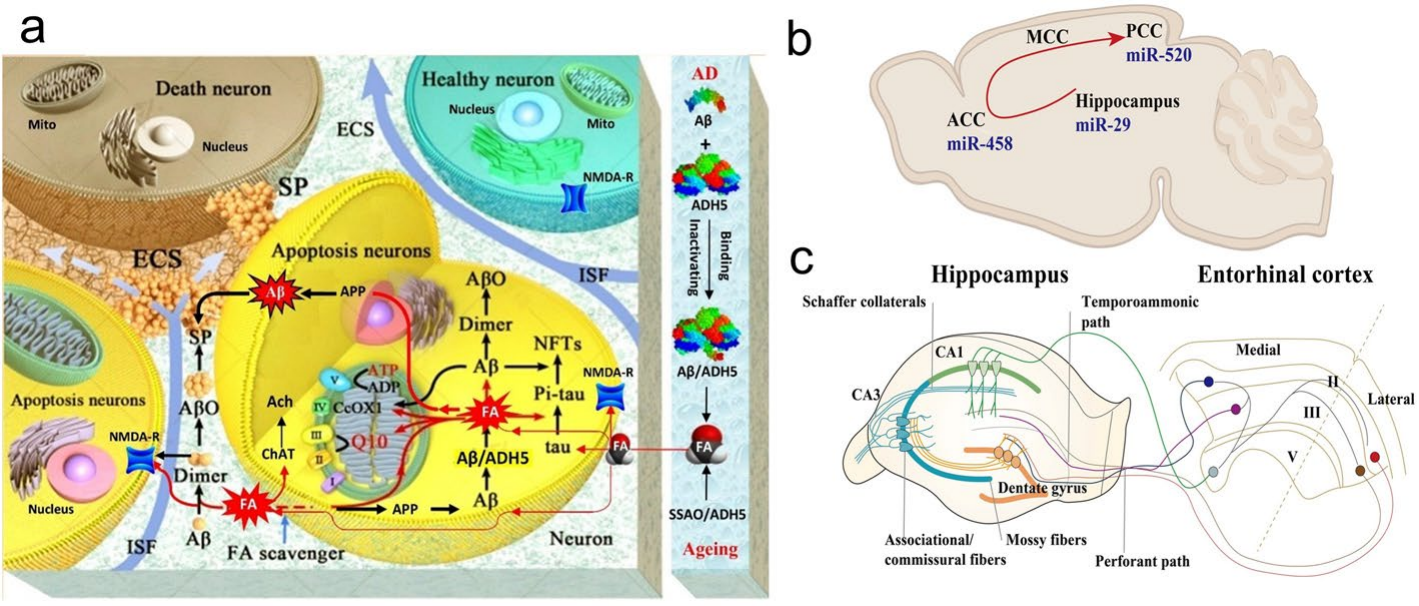
Endogenous formaldehyde (FA) accumulates in various brain areas and is associated with typical pathologies in AD. **a** FA cross-links Aβ, blocking the ECS space; at the same time, it causes excessive phosphorylation of tau protein and participates in neuronal apoptosis [54]. **b, c** FA accumulates successively in the hippocampus and cortex, which is related to the tissue specificity of miRNA distribution and the direction of hippocampal fiber tracts. This may mediate the temporally and spatially sequential accumulation of Aβ [40, 42, 162]

FA may be a key factor in the prion-like sequential deposition of Aβ in the ECS. In APP/PS1 mice, FA is sequentially deposited in the hippocampus, prefrontal cortex, and posterior occipital cortex, which is consistent with the direction of sequential Aβ deposition [40, 42]. FA, an endogenous molecule, is significantly elevated in people over 65 years of age and its level is negatively associated with cognitive function in AD patients. As a classical protein cross-linker, FA not only cross-links with hemoglobin and albumin, which leak from aging cerebral blood vessels, but also participates in the cross-linking of Aβ and hyperphosphorylated tau [54] (Fig. 4a). Age-associated FA cross-links with the lysine 28 residue (K28) in the β-turn of nontoxic Aβ monomer to form toxic dimers, oligomers, and protofibers. Furthermore, oxidative demethylation at groups (-CH2-OH) of two serine residues (S8 and S26) in Aβ42 induces production of FA [159]. This creates an irreversible vicious cycle in the context of obstructed ISF flow. Additionally, latest work also suggested that FA accumulation in AD mouse model might drive sequential Aβ deposition along the hippocampal fiber tracts, potentially via region-specific downregulation of microRNAs [160, 161] (Fig. 4b, c).

This hypothesis still needs to be further integrated with ISF dynamics, the sequential accumulation of FA in the human brain, to account for the specific mechanism of FA in the sequential deposition of Aβ in the ECS.

### Brain lymphatic vessel-like structure

**Fig. 5:**
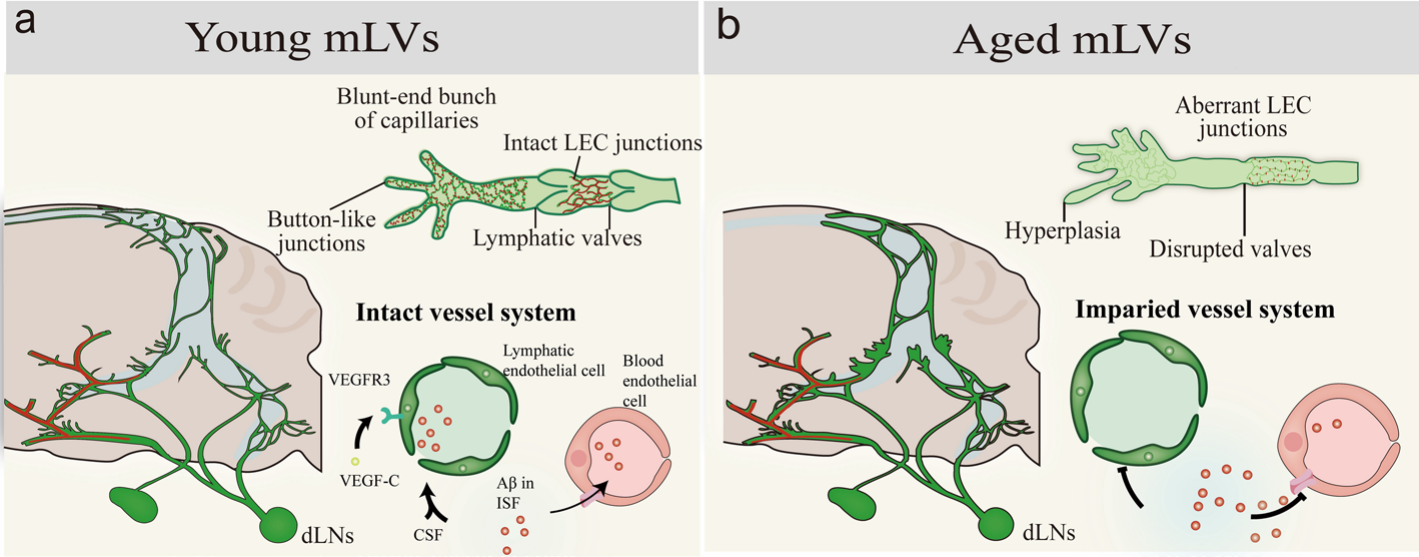
Central-peripheral drainage impairment in aging. **a-b** Basal meningeal lymphatic vessels in young and aged mice

In recent years, the discovery of mLVs (dorsal, basal) and subarachnoid lymphatic-like membranes has broken the old view that the brain lacks lymphatic drainage pathways [163]. They, along with the nasopharyngeal plexus lymphatic vessels, constitute a conduit for the outflow of CSF to the dcLNs [164]. In conditions of aging, inflammation, and AD, damage to the endothelium of these lymphatic/membrane-like structures and recruitment of glial cells result in impaired CSF drainage to the periphery, facilitating the sequential accumulation of core AD proteins, such as Aβ, in the brain and ultimately in the cognition-related cortex, which is involved in the progression of cognitive dysfunction in AD (Fig. 5).

#### Changes in brain lymphatic vessels during normal aging

*mLV-dcLN drainage* In 2015, Kipnis’s group discovered dorsal mLVs in rodent and human brains, serving as conduits between the CNS and the peripheral immune system for CSF drainage [165]. In 2019, meningeal lymphatics with greater drainage capacity were identified in the basal part of the skull. Owing to the presence of a lymphatic flaps and nearby capillaries, basal meningeal lymphatics are more efficient for CSF uptake and unidirectional efflux, representing the primary pathway for CSF drainage and removal to the dcLNs [166].

Both dorsal and basal mLVs show significant plasticity. In aged mice, the basal mLVs show increased branching and enlarged size, which may be associated with impaired CSF drainage. Notably, the set of genes involved in immune and inflammatory responses, phospholipid metabolism, ECM organization, cell adhesion, and endothelial tubular morphogenesis is altered in lymphatic endothelial cells (LECs) of old mice compared to adult-young mice. Age-associated immune and inflammatory responses may drive the mLV plasticity. Acute inflammation induces activation of the VEGF-C/VEGFR3 pathway, which promotes lymphatic vessel dilation and neovascularization and contributes to accelerated immune cell clearance and antigen presentation [167]. Chronic inflammation then triggers structural degradation and dysfunction of mLVs, further exacerbating neuroinflammation. Notably, treatment with recombinant VEGF-C significantly increases the diameter of meningeal lymphatics, showing therapeutic potential [165].

*Nasopharyngeal lymphatic plexus-deep (NLPD)-cervical lymph node drainage* The nasopharyngeal lymphatic plexus is a network of lymphatic vessels located in the nasopharyngeal region. It connects the lymphatic system of the nasal cavity, pharynx and skull base region. These lymphatic vessels are connected to dcLNs and contain specialized valvular structures to ensure unidirectional CSF flow, forming an important lymphatic drainage pathway in the head and neck region [168]. Notably, the CSF outflow through the plexus and medial cervical lymphatic pathways is on average 180% greater than that through the lateral dural lymphatic pathways [169].

The nasopharyngeal lymphatic plexus also undergoes structural and functional decline with age. The nasopharyngeal lymphatic plexus shows ageing-related atrophy, with a 14% decrease in the overall area and a 41% decrease of the number of PROX1⁺FOXC2⁺ lymphatic valves in aged mice (73–102 weeks) compared to adult mice (8–12 weeks) [169]. However, the downstream deep cervical lymphatics were not changed in aged mice. In addition, the nasopharyngeal plexus lymphatic endothelium of aged mice exhibited inflammatory ageing features, and single-cell analysis revealed significant upregulation of type I interferon signaling and inflammation-associated genes such as *Irf7*, *Ifitm2*, and *Zbp1* [167, 169, 170]. Moreover, CSF efflux through this pathway can be enhanced by adrenergic activation or nitric oxide signaling regulation in the lymphatic smooth muscle. However, sustained chronic inflammatory conditions may lead to lymphatic tissue remodeling, impairing its clearance function.

#### Changes in brain lymphatic vessels in AD dementia

The dcLNs are the main bridge for CSF drainage to the periphery, and the drainage efficiency declines with age. Ligation of dcLNs blocks CSF drainage to the dcLNs via mLVs and dcLNs, inhibits peripheral Aβ clearance, and exacerbates AD pathology [171]. Recently, lymphatic-venous anastomosis (LVA) has been performed in China to treat patients with AD. Preliminary results suggest potential improvements in cognitive function and AD-related biomarkers. However, current evidence is limited by small sample sizes, nonrandomized designs, and methodological variability [172].

*Meningeal lymphatic vessel-deep cervical lymph node drainage* AD mLV dysfunction is closely associated with Aβ deposition and represents a significant mechanism contributing to neurodegeneration. The impaired mLV function affects not only CSF influx and efflux but also ISF diffusion, contributing to cognitive decline [167]. Although treatment with mVEGF-C can modulate the function of mLVs and promote CSF efflux, no significant therapeutic effect was found in the 6- to 7-month-old J20 mouse model, suggesting that severe dysfunction of the mLVs may not be present in the early stages of AD.

*NLPD-cervical lymph node drainage* There is still a lack of studies on the nasopharyngeal lymphatic plexus in AD. The changes of NLPD in AD may be similar to those associated with aging. LECs with NLPD in aged mice exhibit increased levels of apoptosis and pathological protein deposition, with a 2.5-fold increase in the expression of phosphorylated tau [169]. The AD-associated neuroinflammatory state can interfere with the immune cell migration and antigen presentation mediated by lymphatic pathways, exacerbating immune imbalances in the brain. Proinflammatory cytokines (e.g., TNF-α and IL-1β) may induce dilatation and increase permeability of nasopharyngeal lymphatic vessels, enhancing immune cell extravasation and migration. Chronic inflammation may lead to remodeling of lymphoid tissues, which affects immune homeostasis [163, 170].

## Effects of light therapy on glymphatic system and Aβ

### PBM therapy

**Fig. 6:**
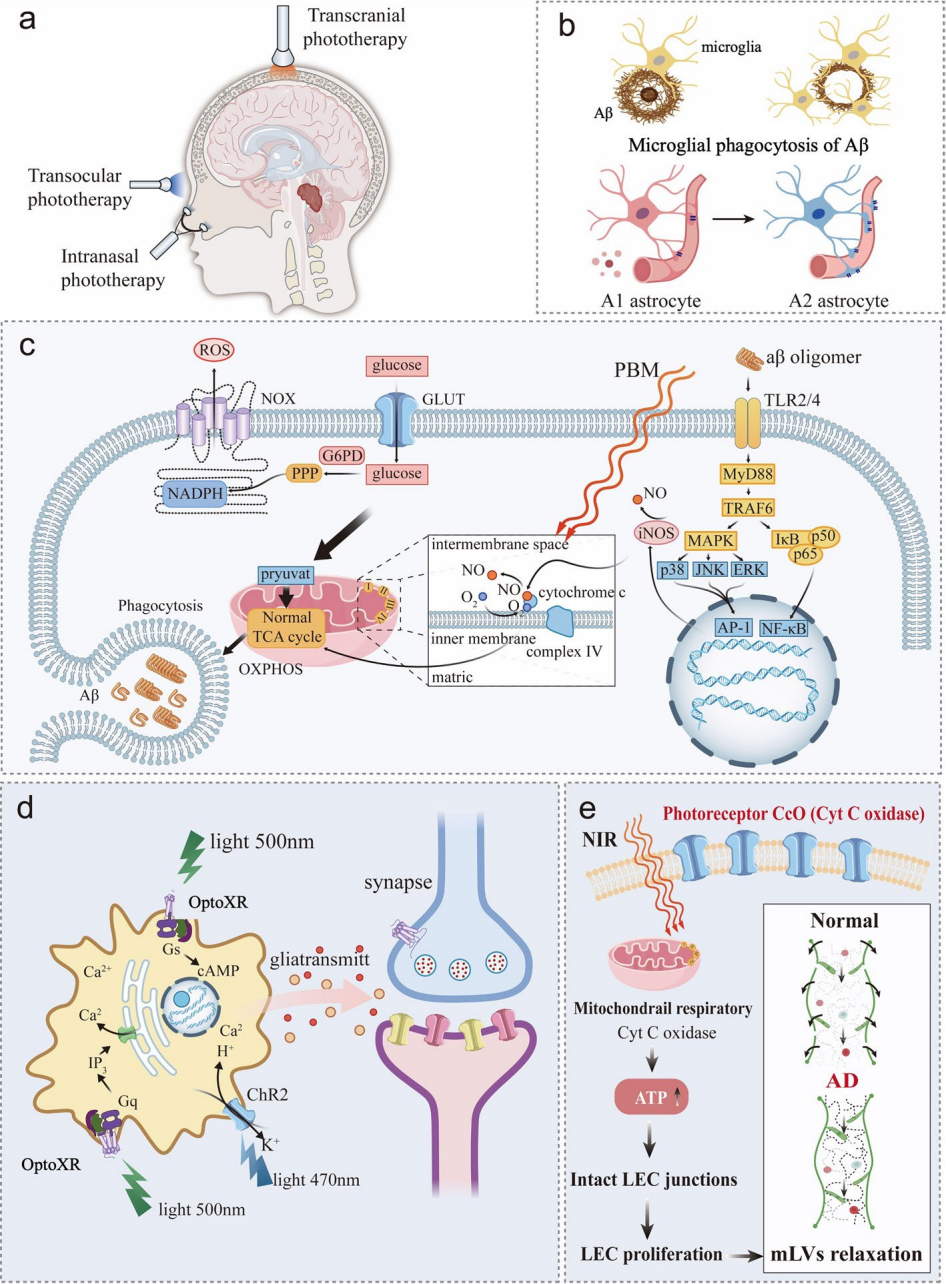
Effects of light therapy on Aβ clearance within the brain. **a** Three different phototherapy approaches. **b** Photobiomodulation regulates morphological changes of glial cells and promotes microglial phagocytosis of Aβ. **c** Photobiomodulation activates cytochrome *c* enzymes in mitochondria to eliminate factors promoting Aβ aggregation. **d** Optogenetic technology regulates astrocytes and restores the synaptic damage of neurons in AD. **e** Near-infrared (NIR) light improves the function of lymphatic endothelial cells in mLVs

PBM is performed with nonionizing light sources, including LED spectroscopy, laser spectroscopy, visible spectroscopy, and infrared spectroscopy (Fig. 6a), with wavelengths ranging from 400 to 1300 nm [173]. For encephalopathy treatment, red light (600–740 nm) and near-external red light (740–1100 nm) [174] have been used, demonstrating efficacy in mouse models of AD and Parkinson's disease (PD). PBM can inhibit the aggregation of Aβ and improve the clearance rate [175], thereby slowing cognitive impairment and memory impairment. However, large-scale clinical studies are currently lacking. Even in animal experiments, results have been inconsistent. For example, Sipion et al. reported that PBM at 810 nm had no effects in improving AD-related cognitive functions [176].

The increased metabolite clearance in the brain by PBM is associated with enhanced glymphatic system function. A possible mechanism is that PBM enhances blood perfusion to increase the influx of CSF in the paravascular pathway, inhibits the deposition of misfolded proteins in the ECS of the brain and restores the drainage of ISF [177]; at the same time, the function of mLVs is enhanced.

#### PBM therapy eliminates Aβ

Light can exert a noninvasive biological regulatory effect on the CNS through thermal and optical effects. Photobiological regulation has been proven to be beneficial for improving the neural functions of the aging brain and enhancing cognitive functions such as attention, learning and memory. Red light/near-external red light of multiple wavelengths significantly improves the cognitive function of AD patients [178]. The key target of PBM is the mitochondrial enzyme cytochrome* c* oxidase (CCO), which plays a role in eliminating ROS and inhibiting inflammation (Fig. 6c) [179]. Continuous irradiation at 808 nm (20 mW/cm^2^) for 6 weeks significantly activated CCO in APP/PS1 mice and increased ATP content in APP/PS1 mouse model [180].

PBM can also improve cognitive function by regulating glial cells and inhibiting the aggregation of Aβ. On the one hand, PBM can promote the transformation of astrocytes and microglia to anti-inflammatory phenotypes (A1 and M2, respectively) and inhibit neuroinflammation at the cellular level, which is also related to the improvement of mitochondrial activity [181]. On the other hand, microglial phagocytosis is one of the main pathways for Aβ clearance (Fig. 6b). Studies have shown that 1070 nm near-external red light (4 J/cm^2^, 10 Hz) improves the cognitive function of 5 × FAD mice by promoting the transformation of microglia to the M2 anti-inflammatory type and alleviating Aβ burden [182]. This is related to the fact that the exosomal Mir-7670-3p secreted by microglia inhibits the expression of activating transcription factor 6 (ATF6), alleviates endoplasmic reticulum stress and protects against neuronal plasticity. Furthermore, long-term exposure to near-external red light at 808 nm leads to recruitment of microglia around amyloid plaques in AD rat model by increasing the expression of IL-3 in astrocytes and IL-3Rα in microglia [183].

Red light also ameliorates neuronal damage caused by Aβ. Continuous 635 nm red light irradiation (6 J/cm^2^) for one month can ameliorate the synaptic dysfunction in cortical and hippocampal neurons of APP/PS1 mice [184] and promote neurogenesis [185]. Similarly, low-level laser therapy at 632.8 nm rescued neuronal loss and dendritic atrophy, potentially by increasing expression of brain-derived neurotrophic factor (BDNF) in Aβ-treated hippocampal neurons and cultured APP/PS1 mouse hippocampal neurons [186].

PBM directly inhibits the conformation of Aβ. PBM can directly change the secondary conformation of tubulin, and when combined with nanoparticles or xanthene, can inhibit Aβ fibrosis. In vitro experiments have shown that near-infrared (NIR) light (810 nm, 10 Hz, 22.5 J/cm^2^) significantly reduces the α-helix content of tubulin and simultaneously increases the β-sheet content [187]. In addition, green light excitation of rose bengal (an xanthene dye) interferes with an early step of the Aβ self-assembly pathway, suppressing the conformational transition of Aβ monomers to β-rich sheet structures. In addition, it not only effectively inhibits the aggregation of Aβ but also reduces the cytotoxicity induced by Aβ [188].

More notably, in vivo studies have demonstrated that PBM significantly reduces Aβ deposition in the hippocampus and cortex, and improves cognitive function in mice by promoting a shift of APP processing toward the non-amyloidogenic pathway. This effect is thought to result from the CCO activation and subsequent PKA/SIRT1 signaling [189].

#### PBM restores ISF and CBF flow

Neurodegenerative diseases such as AD and PD all involve spatial blockage of the cerebral ECS caused by pathogenic protein deposits such as tau, Aβ, and α-syn, which slows ISF drainage. While reducing the aggregation of pathogenic proteins such as Aβ is the most therapeutic goal, clinical trial outcomes have not consistently mirrored Aβ reduction. Restoring ISF drainage, protecting cerebral ECS function, and improving PVS drainage are the new goals of encephalopathy treatment.

PBM improves the cognitive function of AD mice by enhancing waste clearance in the ECS. Red light (755 nm) accelerates the clearance of inflammatory factors in the rat ECS by promoting ISF flow and CSF influx, potentially via restoring AQP4 polarization [190]. Additionally, PBM exerts biological effects by activating many photosensitive proteins to inhibit the formation of Aβ. We found that 630 nm red light energy directly disrupts the Aβ secondary structure and reduces Aβ aggregation in vitro [177]. Red light from a light-emitting diode activates formaldehyde dehydrogenase to degrade FA, reduces Aβ aggregation in the ECS in mouse brain and rescues memory deficits in AD mice [191]. Red light can also activate TRPV1 on microglia, promoting phagocytosis and degradation of α-syn from the ECS in PD mice [192].

CBF is an important factor that regulates the inflow of CSF into the brain parenchyma. Vascular pulsatility drives CSF inflow through paravascular pathways, and CBF correlates positively with the CSF drainage volume. The destruction of blood vessels and surrounding structures in AD reduces CBF, resulting in a reduction in CSF inflow and the accumulation of Aβ. Clinical studies show that red light at 627 nm (70 mW/cm^2^, 10 J/cm^2^) significantly increased the contraction (25%) and relaxation (30%) rates of the human left middle cerebral artery [193], implying improvement in the CBF. However, CSF drainage was not monitored in this study.

#### NIR light increases meningeal lymphatic function

NIR light has a wavelength of 740–1100 nm and can effectively penetrate soft and hard tissues into deep structures. In humans, NIR light in the 10–15 W power range is thought to exert biological effects within 3 cm, despite significant energy attenuation (~ 0.45%–2.90% of 810 nm light penetrated 3 cm of tissue) [194]. Light can still penetrate and reach the important meningeal structures of the brain, such as the dura mater and arachnoid membrane (approximately 16–22 mm from the body surface). This provides the basis for the regulation of mLVs (located in the dura mater of humans and animals) by NIR.

The mLVs drain macromolecules to dcLNs via CSF and play a crucial role in maintaining brain homeostasis [195]. Recent studies have shown that transcranial light therapy at a wavelength of 808 nm effectively restores the distribution and drainage function of mLVs and improves cognitive function in various AD mouse models (5 × FAD, APP/PS1) [196]. The beneficial effects of NIR on the drainage of mLVs depend on the functional regulation of meningeal glymphatic endothelial cells, including enhancing mitochondrial metabolism to preserve cell function, maintaining the intact structure, and compacting arrangement of mLVs. In 4–6 months 5 × FAD mice, 1267 nm NIR promoted the drainage of CSF into the dorsal mLVs [197]. The improved cognitive function by 635 nm red light in AD mice has also been associated with enhanced CSF drainage via the dorsal mLVs [198].

Furthermore, impaired mLV drainage exacerbates the microglial inflammatory response in AD patients, which further disrupts mLV integrity [199]. NIR light has demonstrated anti-inflammatory effects in animal models, significantly inhibiting neuroinflammation mediated by the NF-κB pathway [200]. This may be another potential mechanism by which NIR improves mLV drainage, although further research is needed. Notably, in neurodegenerative diseases, mLVs are involved in pathophysiology together with neuroimmunity and neuroinflammation.

#### Photodynamic therapy (PDT)

In classic PBM therapies (especially visible light and NIR light), there is significant energy attenuation during light penetration through the cranial and brain tissues. This restricts the treatment of early Aβ deposition in deep brain regions. PDT takes redox as the core mechanism and overcomes the limitation of poor penetration of phototherapy [201]. This approach enables photo-oxidization of Aβ aggregates in deep brain regions such as the hippocampus and medial prefrontal cortex, inhibiting their neurotoxicity. Classic PDT employs light-activated small-molecule photosensitizers to generate ROS that inhibit Aβ aggregation or degrade fibrils [202]. Notably, the high H₂O₂ concentrations in the AD pathological microenvironment can directly activate nanoparticles loaded with specific photosensitizers, and further generate singlet oxygen (1O₂) to induce the degradation of Aβ aggregates. This is known as the "in-situ activation-chemical excitation" strategy [203]. Another strategy to improve tissue penetration is to change the structural sequence of Aβ under the light ("molecular light") generated by the chemiluminescent probe ADLumin-4 molecule. Kuang et al. confirmed that combining CRANAD-147 with LED or CRANAD-147 with ADLumin-4 (molecular light) can effectively slow down Aβ accumulation in 5 × FAD mice [204]. From small-molecule photosensitizers to nanoparticles and photodynamic microrobots, PDT continues to evolve toward enhanced tissue penetration and target specificity.

### Photosensitive proteins

Photosensitive proteins are receiving increasing attention for use in AD therapy, especially in modulating neural circuits in deep brain regions. Conventional PBM relies primarily on red or NIR light to irradiate the brain through the scalp and skull. However, the intensity of light is significantly attenuated as it passes through tissues such as the scalp, skull and meninges. Studies have shown that after penetrating the human scalp and skull, 808 nm light actually reaches the cerebral cortex at only 0.2% to 10% of the irradiated energy, depending on the individual's anatomical structure and light parameters [205]. In addition, while some studies have shown that 808 nm light can penetrate 40–50 mm in the brain under specific conditions (e.g., using high-power lasers), such approaches carry thermal damage risks. Consequently, the conventional PBM shows limited efficacy for deep brain regions such as the hippocampus and amygdala—key brain regions of early Aβ accumulation, during critical therapeutic window for AD. In this regard, photosensitive protein technologies (e.g., optogenetics) offer new possibilities for targeted deep brain modulation.

The optogenetic technique enables modulation of specific neuronal activities with higher spatio-temporal resolution than conventional PBM (Fig. 6d) [206]. By expressing photosensitive proteins, such as Channelrhodopsin, in targeted neurons, researchers can use specific wavelengths of light to precisely activate or inhibit neural populations, thereby modulating the relevant neural circuits. The optogenetic technique can also be used to control γ-aminobutyric acid (GABA) receptor-mediated inhibition [207].

Papez et al. suggested that the hippocampal neural circuits are closely associated with Aβ deposition and can serve as an indicator of early AD [208–210]. Mouse models of early AD show situational memory deficits, and optogenetic activation of hippocampal dentate gyrus (DG) engram cells can lead to the recall of situational memories [211]. Moreover, optogenetics can enhance the functional connectivity of memory trace cells in the hippocampus. This can improve the reversal of dendritic spine deficits associated with neural circuit connectivity deficits in AD, thereby enhancing episodic memory and long-term memory. Using optogenetics, Yang et al. confirmed the existence of a neural loop from pyramidal neurons in the entorhinal cortex projecting to parvalbumin interneurons in CA1, and this loop is degenerated in AD mice. In addition, optogenetic activation of the pyramidal neurons improved spatial learning and memory in AD mice by modulating the balance of excitatory and inhibitory synapses in the CA1 circuit [212].

### Multisensory gamma stimulation

**Fig. 7:**
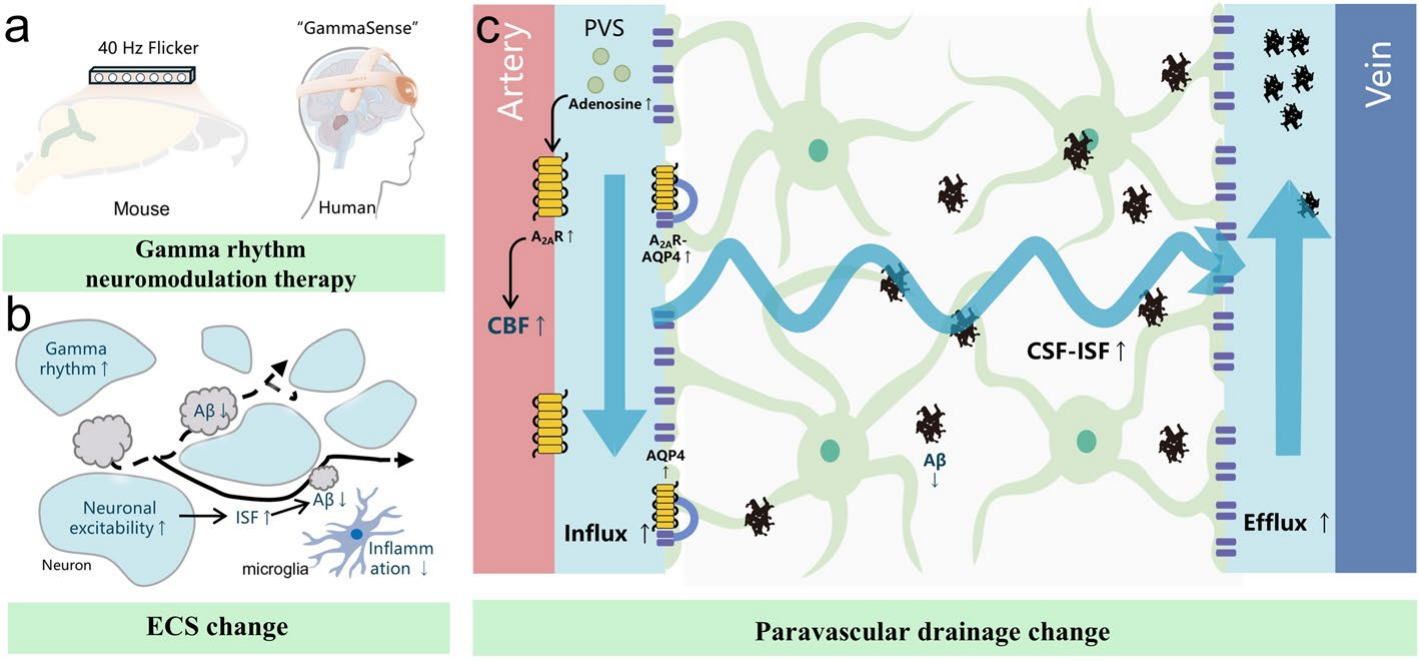
Multisensory gamma stimulation improves AD symptoms via the glymphatic system. **a** Acousto-optic stimulation at 40 Hz has been shown to promote glymphatic system clearance, and has achieved satisfactory efficacy in clinical trials for AD patients. **b, c** Mechanisms have been proposed, such as gamma rhythm oscillation that promotes ISF drainage (**b**) and adenosine receptor activation that promotes CSF-ISF exchange (**c**)

In 5×FAD mice, 40 Hz acousto-optic stimulation promotes glymphatic system clearance (Fig. 7) [8]. Exploration of this specific frequency of 40 Hz originated in 2016, when Tsai et al. reported that the presence of only 40 Hz light stimulation in the γ wave range significantly inhibited Aβ accumulation in the CA1 region of AD mice (5×FAD/PV-Cre) [213]; and acoustic stimulation at 40 Hz exerted a similar effect and affected a wider range of brain regions (including the hippocampus and auditory cortex). More favorable evidence is that the gamma sensory stimulation therapy (40 Hz) has demonstrated efficacy in phase 1/2 randomized controlled, single-blind, multicenter clinical trials, causing 84% and 83% reductions in the ADCS-ADL score and Mini-Mental Status Examination (MMSE) score (assessment of cognitive and memory function) in the treatment group, and a 61% reduction in AD-related brain atrophy and brain volume loss [214].

Opponents argued that 40 Hz light stimulation only caused gamma oscillations in the V1 region of mice, eliciting behavioral aversion [215]. Moreover, although 40 Hz acousto-optic stimulation has been effective in clinical trials, the mechanisms underlying Aβ clearance remains debated. The Tsai team reported that exogenous 40 Hz audiovisual stimulation increased neuronal activity in both mice and humans, promoted CSF-ISF exchange, and enhanced inflow and outflow. This may be achieved by increased cerebrovascular pulses and increased AQP4 polarization [8]. The vasoactive intestinal peptide (VIP), which is released by interneurons under high-frequency stimulation, mediates the cellular processes of lymphoid system clearance (including arterial dilation and astrocyte metabolism). In addition, adenosine and the A2AR receptor (an adenosine receptor) located in cerebral blood vessels, mediate the increase in CBF. A2AR expression is increased, and it is colocalized with AQP4 in astrocytes, which promotes the polarization of AQP4 and the CSF-ISF exchange in mice [216].

### Controversies on non-physical therapies for AD

Recently, LVA, an invasive surgical therapy for glymphatic disorders in AD, has sparked extensive discussions [217]. Proponents claim LVA enhances peripheral lymphatic drainage of CNS-derived waste and improves cognition [218]. This surgery was first performed on a severe AD patient in 2020, with a total of 3 end-to-side/end-to-end anastomoses completed in the bilateral cervical Va lymph node regions. Transient cognitive improvement peaked at one month post-operation, with PET/CT suggesting reduced cerebral amyloid deposition [219]. Since then, multiple small-sample clinical studies have reported the positive effects of LVA treatment in AD patient. However, the LVA surgery, which currently lacks sufficient evidence-based medical data for its safety and effectiveness, has been banned by the National Health Commission of China for AD treatment.

**Fig. 8:**
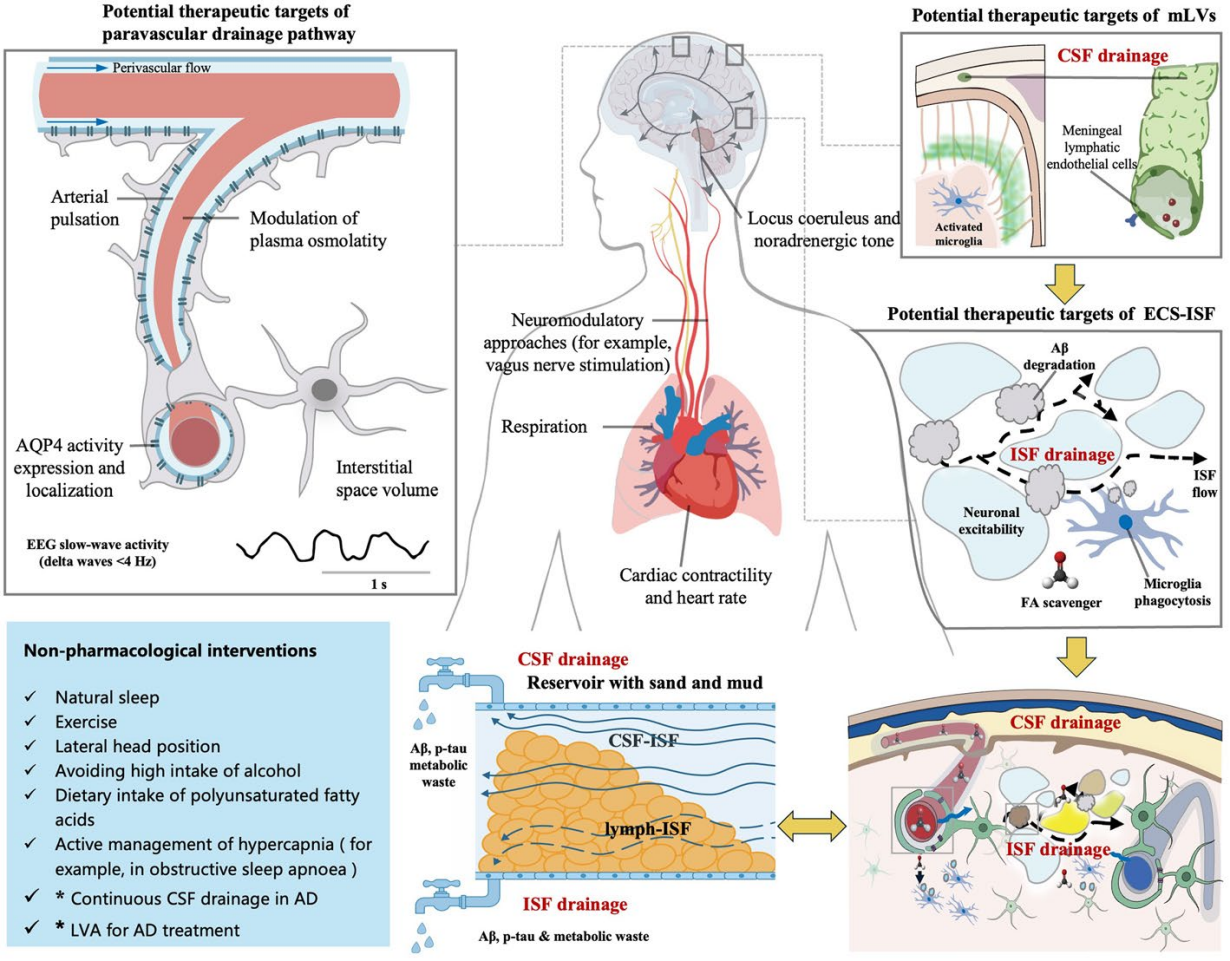
Potential targets and light therapy strategy in the early AD stage. Multiple physical therapies, including phototherapy, and medications, can regulate vascular pulsation, AQP4 polarization, slow-wave activity, etc., in the perivascular drainage pathway. This is the most classic structure and intervention target of the glymphatic system. mLVs contain endothelial cells and immune cells with complex phenotypes and have the potential to become a target for regulating the drainage of CSF to the periphery. In ECS, formaldehyde scavengers effectively improve ISF drainage and cognitive function. In addition, healthy lifestyle habits can also regulate peripheral respiration and heart rate to affect the glymphatic system. Therefore, they are importantnon-pharmacological interventions in the prevention of AD. Finally, achieving rapid drainage of CSF/ISF through surgical operation may also promote the clearance of Aβ in the short term, but controversies remain on the invasive surgical therapy

The most recent prospective single-center study indicated that although subjective reports of nursing staff as well as the MMSE suggest an improvement in patients' cognitive function one month after surgery, important biomarkers such as Aβ42, Aβ40, p-tau, and total tau were not significantly decreased [220]. LVA increases the drainage of glymphatic system to the periphery and also promotes the outflow of ISF in the brain. This will lead to decreased content of ECS/ISF in the brain parenchyma. The convection of CSF-ISF and the diffusion of ISF are further inhibited. Eventually, Aβ and tau cannot be continuously cleared to the periphery, resulting in accumulation. Similarly, continuous CSF shunt has also been proven unable to continuously improve the cognitive function of AD patients: successfully achieving CSF drainage does not ensure continuous clearance of Aβ in the ECS [221]. This can be understood as sediment in a fast-flowing river.

In summary, surgical procedures to improve the function of the glymphatic system and delay Aβ progression represent a promising therapeutic strategy, but these interventions must be based on clear end-point efficacy indicators and a large amount of evidence-based medical data. Currently, although an LVA rat model has been established [222], critical CSF/ISF flow parameters remain uncharacterized. In vivo synchronous monitoring of lymphangion contraction dynamics, flow velocity, and solute transport can be achieved by the 2P-OPTIC (two-photon optical imaging with particle tracking in vivo of CSF tracers) technology, which will provide mechanistic insights to support the clinical translation of LVA [223].

## Potential light therapy strategies in different AD stages

In recent years, development of traditional drugs for the treatment of AD has been challenging. On the one hand, AD pathogenesis involves multifactorial mechanisms, and the clearance pathways of pathogenic proteins such as Aβ remain elusive. On the other hand, the presence of BBB and other ECS partition barriers makes it difficult for traditional drugs to reach deep brain lesion areas. The glymphatic system provides a new therapeutic target for AD. The regulatory mechanisms of therapies targeting the glymphatic system encompass not only regulation of arterial pulse, AQP4 expression, and CBF in the paravascular pathway, but also modulation of neuronal activity, expression levels of transmitter molecules, and the FA concentration in the ECS. Furthermore, they involve the regulation of meningeal LECs, the peripheral respiratory rate, and cardiac contractility.

Considering these challenges, research on phototherapies for AD should include PBM, optogenetics and 40 Hz acousto-optic stimulation, aimed at promoting the glymphatic system clearance (Fig. 8).

There is a consensus that the early stage of AD is the best treatment window. However, core AD proteins such as Aβ begin to accumulate in deep brain regions, such as the entorhinal cortex and the precuneus, which limits the penetrability of mature transcranial phototherapy. The vertical distance from the precuneus to the skull was between 25 and 40 mm, and other brain regions with early accumulation of Aβ have distances between 30 and 50 mm. As described above, transcranial phototherapy has insufficient penetration and energy efficiency in these deep brain regions. Studies have shown that only 2% of 1064 nm laser light can pass through the supraorbital frontal bone of humans [224]. Only 2.9% of the 810 nm light emitted by high-power laser equipment can penetrate 3 cm of scalp, skull or brain tissue [194], accompanied by significantly increased heating effects.

Given the proximity of the entorhinal cortex, precuneus, and related regions to the ventral skull base, nasal phototherapy represents a promising strategy for early intervention [225]. Perforations in the cribriform plate permit direct light delivery to affected regions in early AD. In addition, phototherapy can also act on brain regions such as the hippocampus and amygdala related to cognition and emotion through the prefrontal cortex [226]. In addition, transcranial NIR can promote the drainage of CSF to the periphery through meningeal lymphatic vessels, as does the application of optogenetic technology in the treatment of AD.

Multiple physical therapies, including phototherapy, and medications, can regulate vascular pulsation, AQP4 polarization, slow-wave activity, etc., in the perivascular drainage pathway. This is the most classic structure and intervention target of the glymphatic system. mLVs contain endothelial cells and immune cells with complex phenotypes and have the potential to become a target for regulating the drainage of CSF to the periphery.

## Conclusion

Here, we review glymphatic dysfunction and structural damage across AD stages, proposing the role of the Aβ crosslinker, FA, in the sequential deposition of Aβ. Early AD involves a decline in CSF-based clearance due to aging and sleep disorders, potentially linked to elevated Aβ in plasma and CSF. During progression, factors promoting Aβ deposition, including the accumulation of endogenous FA, further impair the glymphatic system. Upregulation of specific miRNAs may cause the sequential accumulation of FA in the hippocampus, prefrontal cortex, and occipital cortex, resulting in the blockage of the ECS in more brain regions and the stagnation of ISF drainage. Eventually, this leads to irreversible damage to the cognition-related cortical regions.

For early AD (a crucial treatment stage), phototherapies have shown therapeutic potential targeting the glymphatic system. Red light such as 630 nm may improve ISF drainage by removing FA and promoting microglial phagocytosis of Aβ. The 810-nm NIR light can also promote the clearance of Aβ to the periphery by regulating the endothelial cells of mLVs.

Finally, it should be acknowledged that due to the limitations of light penetration and thermal effects, the wavelength selection is a significant challenge in clinical treatment of AD with light therapy. This challenge is of particular importance in early stages of AD—the optimal treatment window. Core markers such as Aβ are deposited in deep brain regions in the early stage. This requires light therapy intervention with higher power and higher tissue penetrability. Moreover, large-scale clinical studies validating light therapy parameters are a prerequisite for clinical application. Crucially, light therapy parameters, such as wavelength, irradiation intensity, energy density, and frequency, have not been fully disclosed and explained in all studies, causing inconsistent outcomes even at identical wavelengths.

## Data Availability

Not applicable.

## Abbreviations

AD: Alzheimer’s disease
CSF: Cerebrospinal fluid
AQP4: Aquaporin-4
Aβ: Amyloid-beta
CBF: Cerebral blood flow
ISF: Interstitial fluid
DTI-ALPS: Diffusion tensor image analysis along the perivascular space
DTI: Diffusion tensor imaging
MRI: Magnetic resonance imaging
BG: Basal ganglia
PVS: Perivascular space
MR: Magnetic resonance
FLAIR: Fluid attenuated inversion recovery
asMTL: Anterior superior medial temporal lobe
DW-EPI: Diffusion-weighted echo planar imaging
sADC: Shifted apparent diffusion coefficient
DCE-MRI: Dynamic-contrast-enhanced MR
CP: Choroid plexus
ECS: Extracellular space
DW-MR: Diffusion-weighted MR
ADC_w_: Apparent diffusion coefficient of water
mLVs: Meningeal lymphatic vessels
IFSH: Inferior frontal sulcus hyperintensity
dcLNs: Deep cervical lymph nodes
SPGR: Spoiled gradient echo
DCE MRL: Dynamic contrast-enhanced MR lymphangiography
PET: Positron emission tomography
FA: Formaldehyde
MCI: Mild cognitive impairment
ROS: Reactive oxygen species
NFT: Neurofibrillary tangle
CNS: Central nervous system
GFAP: Glial fibrillary acidic protein
NREM: Non-rapid eye movement
ECM: Extracellular matrix
LECs: Lymphatic endothelial cells
NLPD: Nasopharyngeal lymphatic plexus-deep
PBM: Photobiomodulation therapy
PD: Parkinson's disease
NIR: Near-infrared
BBB: Blood–brain barrier
